# Parasite Fauna of the White-Streaked Grouper (*Epinephelus ongus*) from the Thousand Islands, Java, Indonesia

**DOI:** 10.1007/s11686-020-00312-0

**Published:** 2020-12-14

**Authors:** Svenja Koepper, S. Nuryati, Harry Wilhelm Palm, S. Theisen, C. Wild, I. Yulianto, S. Kleinertz

**Affiliations:** 1 Marine Ecology, Faculty of Biology and Chemistry (FB 2), University of Bremen, UFT, Leobener Str. 6, Room 2160, 28359 Bremen, Germany; 2 Aquaculture and Sea-Ranching, Faculty of Agriculture and Environmental Sciences, University of Rostock, Justus-von-Liebig-Weg 2, 18059 Rostock, Germany; 3 Department of Aquaculture (S. Nuryati) and Department of Fisheries Resources Utilization (I. Yulianto), Faculty of Fisheries and Marine Sciences (S. Kleinertz), IPB University, Jl. Agatis Kampus IPB Dramaga, Bogor, Indonesia; 4 Wildlife Conservation Society Indonesia Program, Jalan Malabar 1 No 11, Kota Bogor, Jawa Barat 16151 Indonesia

**Keywords:** Fish stock separation, Parasite diversity, *Gyliauchen* cf. *nahaensis*, Grouper fisheries management, Seafood health risks, Food safety of fisheries products

## Abstract

**Purpose:**

Fish parasites can cause diseases in humans and lead to commercial losses in fisheries and aquaculture. The objectives of this study were to analyze *E. ongus*’s parasite fauna regarding food safety and parasite transmission risk between *Epinephelus* species and test whether *E.ongus* populations can be distinguished by their parasite community.

**Methods:**

We studied the metazoan parasite fauna of 30 white-streaked groupers *Epinephelus ongus* from the Thousand Islands, Java Sea, Indonesia, and compared the parasite community with specimens from Karimunjawa archipelago, Java Sea, from a former study. We used common fish parasitological methods for fish examination and parasite calculations.

**Results:**

We found 12 metazoan parasite species, establishing five new host and five new locality records, increasing the known parasite fauna of *E. ongus* by 21%. No anisakid worms infected *E. ongus*. All but one (trematode *Gyliauchen* cf. *nahaensis*) species have been previously reported from *Epinephelus*. Parasite abundance of *E. ongus* differed significantly between the two regions.

**Conclusions:**

Due to a certain degree of host specificity to groupers, there is potential risk of parasite transmission from *E. ongus* into groupers in mariculture or surrounding fishes, which increases (sea) food security related health risks from zoonotic parasites and calls for better monitoring and management plans for *E. ongus*. The regional separation of the Thousand Islands and Karimunjawa with different food availability and fish ecology causes different parasite abundances, distinguishing two separate *E. ongus* populations by their parasite fauna.

## Introduction

With more than 17, 000 islands and 80, 791 km coastline, Indonesia is the biggest archipelago in the world [1]. Consequently, fisheries and aquaculture play an important role for the national economy [2]. In 2016, behind China, Indonesia ranked second in marine capture production with over 6 million tons [3].

Grouper (Serranidae: Epinephelinae) fisheries is of high economic value for food supply and the live reef fish trade in Indonesia, where they are mainly exported to China, Japan and Singapore [4]. Serranids also contribute to stable livelihoods in developing countries and are important for reef ecosystems as (top) predators [5, 6]. A rising demand for serranids increases fishing effort which drives their risk of extinction [7]. It is estimated that 19 grouper species are threatened and especially the live reef food fish trade remains largely unmanaged [7, 8]. Further knowledge on the ecology and life history of these commercially important species is needed to predict future implications of increasingly high fishing pressure.

Parasites are one of the most successful life forms on Earth [9]. They can impair their fish hosts and also human consumers when raw or undercooked fish is consumed, causing zoonosis as a disease transmitted from animals to humans [10]. The most well-known marine waterborne disease is anisakiasis [11] caused by nematodes of the family Ansiakidae. Monitoring parasites of food fishes is important to continuously assess sea food health risks. Previous studies have suggested a low risk of anisakiasis in Indonesian waters [12–14], but climate change may shift marine boundaries causing different food web compositions in the future [15, 16] and there are also other marine helminths with zoonotic potential, e.g., *Hysterothylacium* or *Pseudoterranova* [13]. Diverse ecosystems such as Indonesia have huge potential for parasitological research [14]. Parasites provide new insights into fish diet, habitat range and trophic position [17, 18] and inform about host ecology due to different infection pathways or accumulation of pollutants [19, 20]. Consequently, commercially important and often aquaculture species such as the groupers *Epinephelus coioides* and *E. fuscoguttatus* have been subject to a number of disease, parasite and environmental studies [19–26].

Today, fishing pressure is shifting toward smaller species such as *E. ongus*, which is now targeted as food fish in some regions, e.g., Japan [27, 28]. Demand for *E. ongus* has increased steadily [28, 29], also based on landings of *E. ongus* in Indonesia (Karimunjawa) [28, 30]. With only seven previous studies on the parasite fauna of *E. ongus* in South-East Asia and Australia [28, 31–36], we herewith sample this fish species from the Thousand Islands archipelago in close proximity to the heavily polluted Jakarta Bay. Our objectives for this study are to assess the transmission risk of parasites from *E. ongus* to cogeners and vice versa and to determine parasite-borne health risks of *E. ongus* to consumers in terms of food safety and to mariculture fish. Additionally, we aim to test whether *E.ongus* populations can be distinguished by their parasite community. For that we compare our data to a former study where the parasite fauna of white-streaked groupers (similar in size and weight) was analyzed in Karimunjawa, an archipelago off the coast of Java, Indonesia, in 2013 by Neubert et al. [28] and discuss infection patterns, parasite biodiversity and fish ecology data.

## Materials and Methods

### Sample Collection

**Fig. 1:**
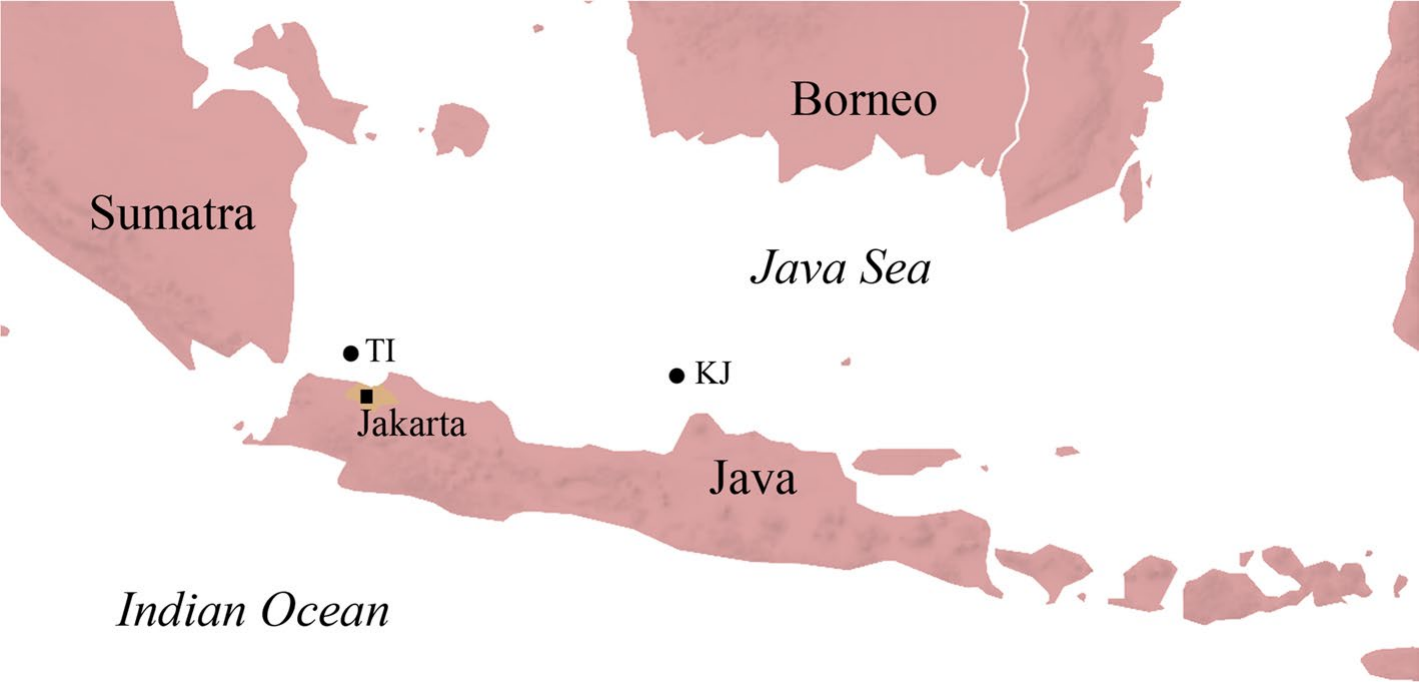
Map of study areas on Java, Indonesia. *TI* Thousand Islands, *KJ* Karimunjawa (samples taken in 2013 by Neubert et al. [28])

From April to June 2018, a total of 30 spearfished *Epinephelus ongus* were obtained from fishermen in the Thousand Islands archipelago (5°48′07.7′′S 106°30′38.4′′E), north coast of West Java, Indonesia (Fig. 1; Table 1). Fish were individually frozen in plastic bags, usually for 2–5 days, until examination inside the laboratory. Fish were defrosted in lukewarm water and morphometric data were taken (total length, standard length, total weight and gutted weight). Fluids from inside the plastic bags were examined individually under the stereo microscope (Zeiss, Stemi DV4).

**Table 1:** Fish biological parameters of *Epinephelus ongus* from the Thousand Islands and Karimunjawa

Location	TL [cm]	SL [cm]	TW [g]	SW [g]	m	f	n.i
Thousand Islands	24.0 ± 2.8 (20.2–30.8)	20.5 ± 2.4 (16.9–26.6)	259.7 ± 99.5 (137.1–525.8)	241.6 ± 92.8 (127.3–494.2)	6	24	–
Karimunjawa^a^	25.5 ± 2.5 (20.5–30.4)	21.8 ± 2.3 (17.0–26.2)	266.3 ± 90.1 (108.5–498.0)	238.0 ± 80.5 (99.8–463.6)	23	7	5

*SL* standard length, *SW* slaughter weight, *TL* total length, *TW* total weight, $\overline{x}$ ± *SD* mean ± standard deviation, range in parantheses, *f* female, *m* male, *n.i.* not identified

^a^Data published by Neubert et al. [28] (sampled in May 2013, using the same methods as described for this study)

### Parasitological Examination

The body cavity was opened and internal organs were removed for subsequent endoparasite examination under the stereo microscope. Fish fillets were examined with translucent light under the stereo microscope. All detected parasites were cleaned from host tissue and collected in 70% ethanol. Selected specimens were transferred into glycerin on microscopic slides following Riemann [37]. Parasites were identified according to keys and original descriptions. After identification, the parasite specimens were deposited at LIPI, Science Centre, Jakarta-Bogor, Indonesia (Accession numbers: 45, 173–181, 542, 1482–1484).

### Processing of Parasitological Data

Parasitological parameters prevalence (P), intensity (I), mean intensity (mI) and mean abundance (mA) followed Bush et al. [38]. The Shannon–Wiener diversity index [39] and Pielou index of evenness [40] were used to estimate the diversity of the metazoan parasite fauna, as well as the Berger–Parker index of dominance [41]. These indices were calculated for ecto- and endoparasites pooled, and endoparasites exclusively [22, 25, 26]. Ecto- to endoparasite ratio was calculated following Rückert et al. [42].

### Statistical Analyses

All data, including the raw data by Neubert et al. [28], were analyzed using PRIMER software (version 7.0) to compare the parasite fauna of *E. ongus* sampled in this study in the Thousand Islands and sampled by Neubert et al. [28] in Karimunjawa. A total of 65 fish were compared and a similarity matrix was calculated by applying a Bray–Curtis similarity measure. The data for the Bray–Curtis index were transformed (fourth root = √√) prior to analysis. Fish without parasites were omitted from the analysis. A multivariant scaling plot (MDS) was used to display the relation between samples based on similarity matrices. The analysis of similarities (ANOSIM) was used to calculate the level of significance between both data sets. The differences between or within the data groups were given by the *R* value that ranged from − 1 to 1. An *R* value of 0 indicated no differences. A value of − 1 meant that the identical data point was located outside of the group, whereas with an *R* value of + 1 they were located within the group. The significance level was stated to *p* < 0.05. To identify which parasite taxa were responsible for the differences between the examined groups, SIMPER was applied. Additionally, a one-way ANOVA with Student’s *T* test (SigmaPlot Version 11.0) was conducted to determine statistical differences in size of the fish between Karimunjawa [28] and the Thousand Islands.

## Results

### Parasite Community

A total of 12 parasite taxa infested *E. ongus* from the Thousand Islands. Four Crustacea taxa [*Alcirona* sp. (Hansen, 1890), *Caligus acanthopagri* (Lin, Ho and Chen, 1994), Gnathiidae indet. (Leach, 1814), Isopoda indet. (Latreille, 1817)], four Nematoda taxa [*Hysterothylacium *sp. (Ward and Magath, 1917), *Philometra epinepheli* (Dewi and Palm, 2013), *Philometra* sp. (Costa, 1845), Nematoda indet.], three Digenea taxa (*Cainocreadium epinepheli* (Durio and Manter, 1968, *Gyliauchen* cf. *nahaensis* [Ozaki, 1937, *Prosorhynchus* sp. (Odhner, 1907)] and one Monogenea taxon [*Pseudorhabdosynochus* sp. (Yamaguti, 1958)] were found.

Ectoparasites represented the most predominant parasite group in *E. ongus.* The isopods *Alcirona* sp. and Gnathiidae indet. had the highest prevalences of 50.0% and 43.0%. Albeit a lower prevalence (23.3%), the Monogenea *Pseudorhabdosynochus* sp. had the highest mean abundance (5.4) and mean intensity (23.0) (Table 2). Fish which served as a host for this monogenean were highly infested (up to 83 worms per fish, Table 2).

**Table 2:** Parasites from 30 specimens of *E. ongus* from the Thousand Islands sampled in 2018, including prevalence (P (%)), intensity (I), mean intensity (mI), mean abundance (mA), and diversity indices

Parasite/parasitological index	Stage	P (%)	mI	(I)	mA
Ectoparasites					
* Pseudorhabdosynochus* sp. (M)***	Adult	23.3	23.0	2–83	5.4
* Alcirona* sp. (Cr)***	Adult	50.0	2.1	1–7	1.0
* Caligus acanthopagri* (Cr)*	Adult	16.7	1.0	2	0.2
Isopoda indet. (Cr)	Adult	13.3	1.0	1–3	0.1
Gnathiidae indet. (Cr)***	Larval	43.3	1.4	1–3	0.6
Endoparasites					
* Cainocreadium epinepheli* (D)*	Adult	10.0	4.3	1–8	8.0
* Gyliauchen* cf. *nahaensis* (D)**	Adult	16.7	1.6	1–4	0.3
* Prosorhynchus* sp. (D)*	Larval	6.7	1.0	1	0.1
* Hysterothylacium* sp. (N)***	Larval	20.0	1.5	1–3	0.3
* Philometra epinepheli* (N)***	Adult	3.3	1.0	1	0.0
* Philometra* sp. (N)*	Adult	10.0	1.3	1–2	0.1
Nematoda indet. (N)***	Larval	3.3	2.0	1	0.1
Parasitological indices					
Shannon–Wiener index of species diversity (total)		1.41			
Shannon–Wienerindex of species diversity (endoparasites)		0.54			
Berger–Parker index of dominance (total)		0.62			
Berger–Parker index of dominance (endoparasites)		0.33			
Pielou index of eveness (total)		0.57			
Pielou index of evenness (endoparasites)		0.28			
Ecto- to endoparasite ratio		0.71			

*Cr* Crustacea, *D* Digenea, *M* Monogenea, *N* Nematoda

*New host records for *E. ongus*

**New host record for epinephelids

***Also found in Karimunjawa (Neubert et al. [28])

**Fig. 2:**
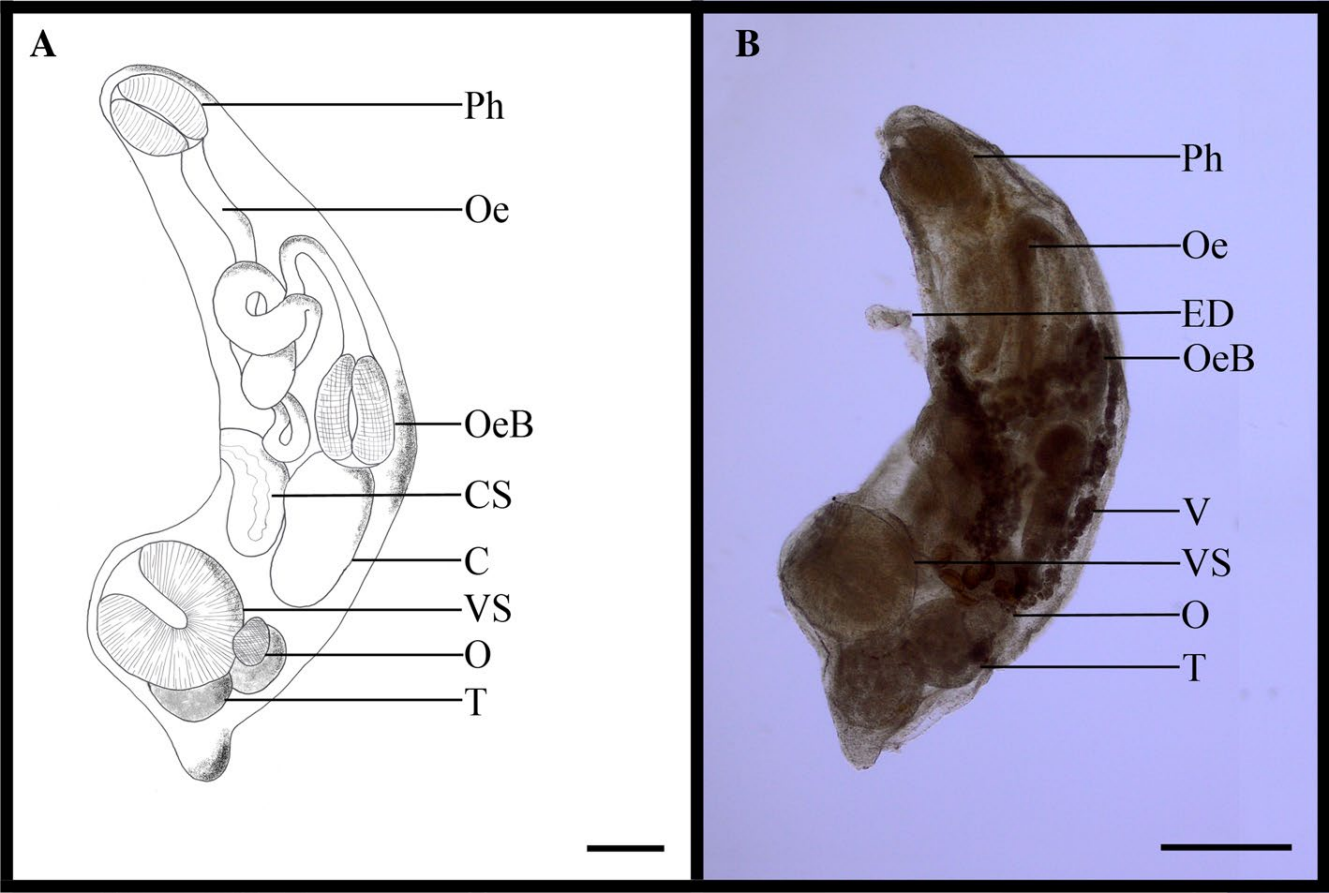
Habitus of two specimens of *Gyliauchen* cf. *nahaensis*. Scale bars **a** 0.2 mm, **b** 0.25 mm. *C* caeca, *CS* cirrus sack, *ED* ejaculatory duct, *Oe* esophagus, *OeB* esophagus bulb *O* ovary, *Ph* pharynx, *T* testes, *V* vitellarium, *VS* ventral sucker

With regard to endoparasites, the nematode *Hysterothylacium* sp. was the most common taxon with a prevalence of 20%, followed by the digeneans *Gyliauchen* cf. *nahaensis* and *Cainocreadium epinepheli* with prevalences of 16% and 10%, respectively. The digenean *Ca. epinepheli* was the most abundant taxon in the present study (see Table 1). *Gyliauchen* cf. *nahaensis* (Table 3; Fig. 2), usually host specific to herbivorous fish (43), is herewith reported for the first time from an *Epinephelus* species.

**Table 3:** Measurements of *Gyliauchen* cf. *nahaensis* from *Epinephelus ongus* from the Thousand Islands compared to data published by Yamaguti [55]^a^ and Nahhas and Wetzel [59]^b^

	*Gyliauchen* cf. *nahaensis*		*Gyliauchen nahaensis*^*a*^		*Gyliauchen nahaensis*^*b*^	
Host	*Epinephelus ongus*		*Acanthurus* sp.		*Siganus punctatus*	
Site of infection	Gills		Intestine		Intestine	
Locality	Thousand Islands, Indonesia		Makassar, Indonesia		Suva, Fiji	
Physiological feature	mm	Percent of body length	mm	Percent of body length	mm	Percent of body length
Body L	0.94–1.67		1.65–2.40		1.30–2.45	
Body W	0.28–0.56	30.3	0.58–0.75	32.8	0.70–1.13	48.8
Oral sucker L	0.14–0.24	14.5	0.13–0.19	7.9	0.20–0.28	12.8
Oral sucker W	0.11–0.18	10.6	0.10–0.15	6.2	0.17–0.24	10.9
Pharynx L	0.14–0.22	13.6	0.15–0.25	9.9	0.22–0.39	16.3
Pharynx W	0.11–0.18	10.5	0.13–0.30	10.6	0.18–0.28	12.3
Ventral sucker L	0.18–0.32	18.5	0.25–0.36	15.1	0.27–0.43	18.7
Ventral sucker W	0.17–0.32	18.8	–	–	0.26–0.45	18.9
Ovary L	0.07	5.7	0.06–0.11	4.2	0.08–0.20	7.5
Ovary W	0.06	4.4	0.06–0.10	4.0	0.07–0.15	5.9
Eggs L	0.06–0.07	5.2	0.08	4.0	0.06–0.08	3.7
Eggs W	0.03–0.04	2.7	0.04–0.05	2.2	0.04–0.06	2.7
Testes L	0.21–0.23	16.9	0.11–0.23	8.4	0.18–0.33	13.6
Testes W	0.21–0.21	16.3	0.10–0.20	7.4	0.13–0.33	12.3

Two taxa of *Philometra* were isolated from *E. ongus, Philometra epinepheli* (from the operculum) and *Philometra* sp. 1 (subcutaneous inside the mouth). The latter might represent a new species. It differed from already recorded *Philometra* species from groupers in Indonesia with two lip-like structures at the mouth, a distinctively shaped swollen esophagus, a different body length and site of infection. So far, only three *Philometra* species have been recorded from *E. ongus*: *P. epinepheli*, *P*. cf. *lateolabracis* and *P. ocularis* [28]. *Philometra* sp. 1 differs from *P. epinepheli* by larger gravid females (280 mm vs. 70–190 mm) and a different infection site (subcutaneous at mouth vs. subcutaneous at operculum) [22, 28, 44]. *Philometra* sp. 1 differs from *P. lateolabracis* in morphology (shorter uterus, ovaries do not reach up to the nerve ring) and in infection site (subcutaneous at mouth vs. gonads) [28, 45–47]. *Philometra* sp. 1 differs from *P. ocularis* by the lack of four large fleshy anterior papillae, a round mouth and in infection site (subcutaneous at mouth vs. eye cavity) [28, 48]. The herewith recorded *Philometra* sp. 1 shares most morphological characters (ovaries not reaching up to nerve ring, similar body length of 240 mm, comparable swollen esophagus) with *Philometra* sp. found by Kleinertz [22] in *E. coioides* and *E. areolatus*, and by Rückert [21] in *E. coioides* and *E. fuscoguttatus*. In Kleinertz [22] *Philometra* sp. 2 infested the gonads, gills, gill cavity, mesentery, mouth cavity, nose cavity and opercula, whereas in Rückert [21] *Philometra* sp. 3infested the gills, gill cavity, mouth cavity and nose cavity. In both previous studies, *Philometra* sp. 2 and 3 were not found subcutaneous at the mouth.

The Berger–Parker index of dominance reached 0.62 when considering the total parasite fauna, but decreased to 0.3 for endoparasites only. The total parasite fauna had a high Shannon–Wiener index of diversity of 1.41. For only endoparasites, the Shannon–Wiener index decreased to 0.54. This trend goes along with a Pielou index of evenness, which was 0.57 including the ectoparasites and decreased to 0.28 for only endoparasites.

### Comparison of Epinephelus ongus from the Thousand Islands and Karimunjawa

**Fig. 3:**
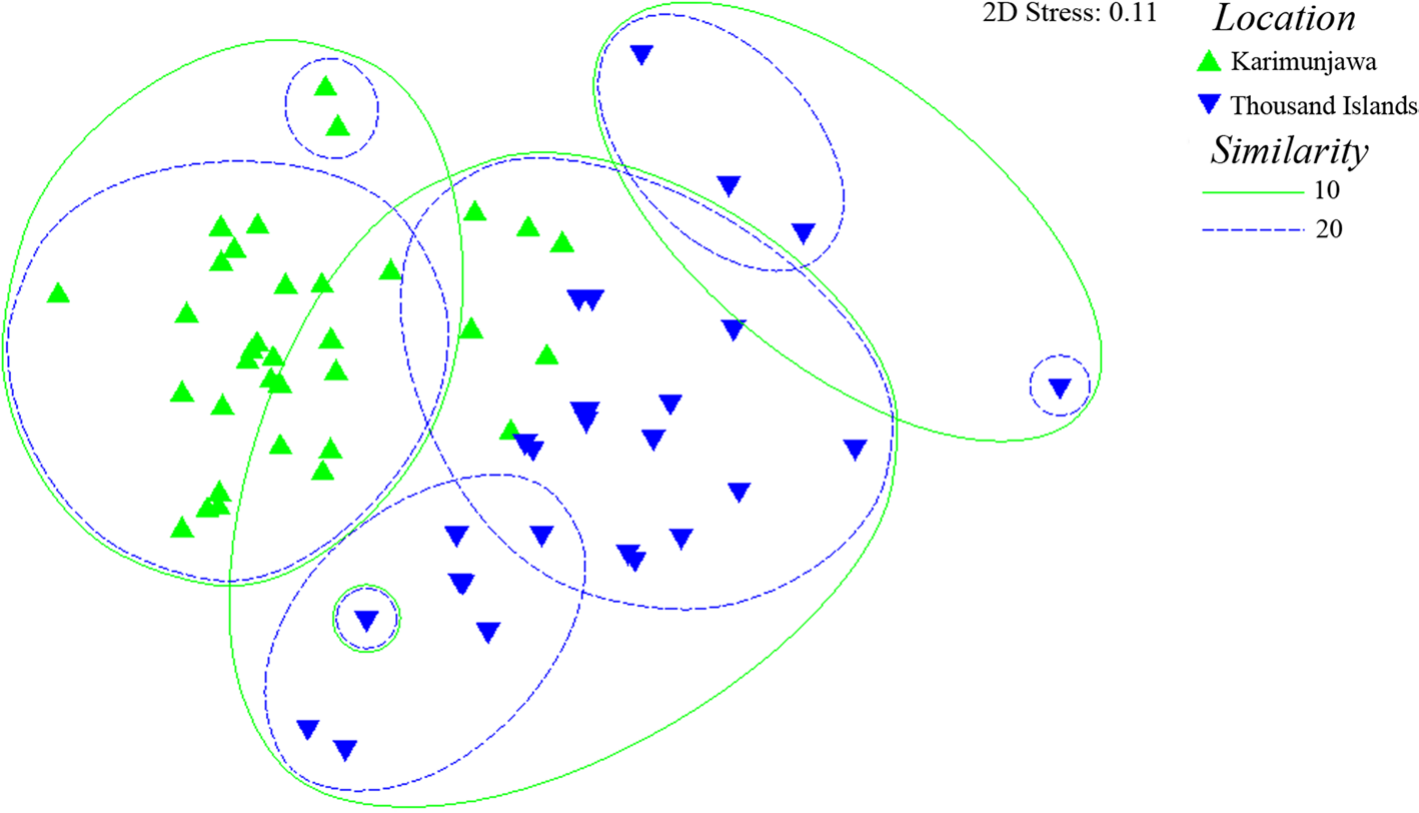
MDS plot of the parasite fauna from *Epinephelus ongus* in Karimunjawa and the Thousand Islands based on parasite abundances. Data from Karimunjawa according to Neubert et al. [28]

A combination of both data sets resulted in a total of 23 parasite taxa (17 in Karimunjawa and 12 in Thousand Islands) for *E. ongus* from Indonesia, 6 of them recorded for both locations (Table 2). The MDS plot based on parasite abundances shows that the infection patterns at both localities were distinctively grouped (999 permutations) (Fig. 3). The data support a significant difference between both sampling sites (ANOSIM: *R* = 0.527, *p* < 0.05), although some samples of Karimunjawa and the Thousand Islands overlap. The major taxa contributing to the separation of the two localities were the gill monogeneans *Pseudorhabdosynochus quadratus* and the crustaceans *Caligus* sp. found in Karimunjawa (SIMPER Analysis).

## Discussion

### Parasite Records

The present study extends our knowledge on the parasite fauna of *E. ongus* by 21%. Five new host and five new locality records were established, with the digeneans *Cainocreadium epinepheli* and *Prosorhynchus* sp., the nematode *Philometra* sp. and the copepod *Caligus acanthopagri* reported for the first time from *E. ongus* and the digenean *Gyliauchen* cf. *nahaensis* for the first time in *Epinephelus*. Additionally, the Thousand Islands represent a new locality record for *G*. cf. *nahaensis*, *Philometra epinepheli*, *Philometra* sp., *C.* *acanthopagri* and *Ca.epinepheli*. To date, seven earlier studies investigated the parasite fauna of the white-streaked grouper, two without any parasite records [32, 36], three reporting one parasite species [31, 33, 35] and two from Indonesia with 17 and eight taxa, respectively [28, 34]. Though Stolz [34] focused on ectoparasites only, the latter findings suggest that the parasite load in white-streaked groupers from Indonesia underlies considerable variability in the number of isolated parasite species.

Ectoparasites predominated the parasite fauna of *E. ongus* and the most abundant and prevalent one was the monogenean *Pseudorhabdosynochus* sp. Parasites of this genus are usually host specific and very common in groupers [49] with *P. quadratus* having been described from *E. ongus* from New Caledonia in the past [33]. The high infestation of these diplectanid monogeneans coincides with findings of other studies [28, 33, 34]. Another generalist was the ectoparasite *Alcirona* sp. which has already been recorded from, e.g., *E. areolatus, E. coioides, E. fuscoguttatus, E. quoyanus* and *Variola albimarginata. Caligus* spp. also infected *E. areolatus, E. coioides, E. corallicola, E. quoyanus* and *V. albimarginata* in Indonesia [28, 34]*.*

Most of the isolated endoparasites have already been found in other serranids in Indonesian waters, e.g., *Ca. epinepheli*, *Philometra* spp., *Hysterothylacium* sp. or *Prosorhynchus* sp. [19–22, 25, 26, 34]. With three *Philometra* species so far isolated from *E. ongus*, this genus is also present in *E. areolatus, E. coioides and E. fuscoguttatus* and commonly infects groupers [28, 50]. In addition, the record of *Philometra* sp. that is suggested to represent a new species and awaits description adds to this biodiversity in groupers [21, 22, 44]. New specimen collections from *E.ongus* are required to confirm its real identity.

Here, similar to earlier studies of *E. ongus*, we did not find any anisakid nematodes and only Neubert et al. [28] reported *Anisakis typica* at a low prevalence of 2.9%. No nematodes infected the musculature, where the possible parasite transmission of zoonotic helminths to the consumer takes place. However, post-mortem migration of helminth larvae into the fillet can occur and *Hysterothylacium* sp. has caused zoonoses in the past, even though it originally infected the intestinal tract of host fishes [51]. With *Hysterothylacium* sp. larvae present in the studied white-streaked groupers, parasite-borne health risks should not be underestimated, but further studies are needed to better understandand evaluate the infection patterns of zoonotic parasites of *E. ongus*, which is a species of rising commercial interest.

With many parasite species having high host specificity to groupers, but a low host specifictiy within epinephelids, there is a risk of parasite transmission between *E. ongus* and its cogeners. This is problematic in and around mariculture facilities, especially under consideration of increasing mariculture activities throughout the region. Consequently, the recorded parasite taxa in this non-cultivated grouper species might still lead to future economic losses [52, 53] in maricultured fish and also impacting wild fish stocks.

### Ecological Parameters

The parasite fauna was predominated by the ectoparasite *Pseudorhabdosynochus* sp., implied by a Berger–Parker index of 0.62 for the total parasite fauna and a lower index of 0.3 for only endoparasites. Evenness and diversity indices were higher in the total parasite fauna compared with only endoparasites. Consequently, the taxa are more evenly distributed even when ectoparasites (including the dominant *Pseudorhabdosynochus* sp.) are considered. This can be explained from a relatively low amount of isolated endoparasites in *E. ongus* resulting in a very low Shannon–Wiener index of 0.54, compared to other grouper species (*E. coioides* up to 1.84, 20, *E. fuscoguttatus*). Both larger sized species have been sampled in more detail with up to 420 specimens per study e.g., [21, 22].

Differences in diet and habitat most likely result in the observed less diverse parasite fauna of *E. ongus* compared to other species [20, 24, 26]. Though diet and behavior of adult *E. coioides, E. fuscoguttatus* and *E. ongus* are approximately the same [54], the parasite composition is different [23, 24, 28]. Sampled specimens of *E. coioides* and *E. fuscoguttatus* often were juveniles, whereas our *E.* *ongus* were adults. Juvenile *E. coioides* and *E. fuscoguttatus* prefer more shallow waters and live over muddy bottoms, fine sediments or in seagrass beds [54, 55], while adult *E. ongus* live in deeper coral reef habitats and rocky bottoms, often in caves in up to 60 m depth [27, 55]. The different ecology, size and age of juvenile and adult *E. coioides* and *E. fuscoguttatus* favor a broad accumulation of parasites, which leads to a diverse and species-rich parasite fauna. Until 2016, 57 different parasite species have been recorded in, e.g., *E. coioides* [22, 26], and even from highly polluted regions around Jakarta Bay up to 31 parasite species were found [20]. This differs from *E. ongus*, with only 30 recorded parasite species so far. Neubert et al. [28] isolated only 17 species in *E. ongus* in Karimunjawa, a region where the biodiversity is believed to be particularly high [28, 29], suggesting that the observed differences in parasite diversity are real and not caused by biased sampling.

An interesting finding was the digenean *Gyliauchen* cf. *nahaensis* in *E. ongus* (Gyliauchenidae, Fig. 2; Table 2). This genus has been recorded in Indonesia only three times, in Sulawesi and Bali [14, 56] in herbivorous reef fish. Gyliauchenid trematodes infect the intestine of their hosts in the Indo-West Pacific [57] and have almost exclusively been recorded from fishes from the herbivorous families Acanthuidae, Chaetodontidae, Pomacanthidae, Scaridae, Siganidae and Zanclidae [43, 58]. However, Srivastava [59] described the species *G. ozakii* from the intestine of the carnivorous predator *Harpodon nehereus* in the Arabian Sea [60]. Lopez [61] isolated *Gyliauchen* sp. from the carnivorous grouper *Plectropomus leopardus* in the Philippines. Here, the finding of *G.* cf. *nahaensis* in the gills of carnivorous *E. ongus* likely depicts an accidental infection, because this genus is very host specific and highly adapted to herbivorous fish [43]. Nevertheless, *G*. cf. *nahaensis* is a rare parasite in Indonesian waters and our findings reflect parasitogical research potential in this region.

### Regional Differences

The herewith analyzed fish specimens from the two archipelagos were similar in size and weight (Table 2). Size is an important factor for comparability because larger fish often have more endoparasites [26]. The fish from the two locations differed in sex ratio (Table 2, more males in Karimunjawa, more females in the Thousand Islands), but there is no consistency in the literature how or if sex affects parasite abundance in groupers. Samples were obtained 5 years apart: Karimunjawa in 2013 [28] and Thousand Islands in 2018. While pollution and anthropogenic effects can have an impact on the fish parasite fauna of groupers [20], previous studies in Indonesia have shown that seasonal change and natural inter-annual variability seem to have low impact on the metazoan fish parasite fauna [23, 26]. Therefore, we conclude that the samples were comparable and we suggest that the significant differences in the parasite abundances of *E. ongus* seen in the MDS plot (Fig. 3) are mainly caused by different biotic and abiotic conditions at both sites and are not biased by annual change or host factors.

While the MDS plot shows a clear separation of samples, some fish from Karimunjawa and the Thousand Islands are grouped together. Most likely this is because these samples harbored identical parasite species, as the locations share six common parasite species. This minor overlap in parasite abundance is not very surprising because we sampled the same species in a quite similar habitat—but it is all the more interesting to observe a significant difference in infection patterns in white-streaked groupers based on the sample location. The ectoparasites *Pseudorhabdosynochus quadratus* and *Caligus* sp. from Karimunjawa were the main factors for the discrepancies. Some gill parasites are thought to accumulate over time, depending on the age of the fish [28]. However, no significant differences in the total fish length between both sites could be detected (Student’s *T* test: *p* = 0.719). Despite high monogenean infestation, Karimunjawa is considered a healthy marine ecosystem due to high endoparasite diversity, high diversity indices and low ecto- to endoparasite ratios [28, 42]. While both localities are important spawning and nursery grounds for groupers [62, 63], Karimunjawa has established management plans, fishing guidelines and protected zones for grouper [29]. Grouper abundance might be higher there which might result in higher ectoparasite transmission and lead to higher ectoparasite infestation in this region.

*E. ongus* from Karimunjawa harbored one single Digenea species, whereas *E. ongus* from the Thousand Islands harbored three. The first intermediate hosts of this parasite group are gastropods. Yulianto [64] reported a sharp decrease of clams in Karimunjawa between 2005 and 2009, possibly linked to increased water pollution from coastal development [65] which may have also affected gastropod abundance. In Jakarta Bay, mollusk abundance decreased over seven decades due to anthropogenic pollution [66]. Finding three digenean taxa in the Thousand Islands suggests that pollution diluted from Jakarta Bay toward the more remote Thousand Islands [66], resulting in a sufficient gastropod abundance for the parasites to complete their life cycles.

## Conclusion

The present study suggests that the parasite fauna of the small-sized white-streaked grouper has less species diversity compared to its larger sized cogeners. Its parasite community differed between the Thousand Islands and Karimujawa archipelagos shown in a comparison between samples from 2013 by Neubert et al. [28] and 2018. This is due to the differences in habitats and regional differences, leading to different ecological needs of the fishes which implies that the parasite composition of *E. ongus* can indicate different populations at the two sites. Fishing efforts on *E. ongus* is steadily increasing and threatens their occurrence throughout the Indonesian archipelago in recent years. This study suggests that the fish parasite community can indicate different origin of groupers and possibly distinct populations, supporting the development of better monitoring and management plans for this species in future. Finding numerous new host and locality records and parasites with a certain affinity to groupers, we suggest potential risks of parasite transmission within different grouper species, for example those cultivated in mariculture facilities or free living groupers. While this poses food safety concerns to consumers of fisheries and aquaculture products in Indonesia, increasing fishing efforts on *E. ongus* call for more research on this grouper species and better monitoring and management plans are needed.

## Data Availability

If wanted, we can share our raw data.
